# Distinct DNA Methylation Dynamics of Spermatogenic Cell-Specific Intronless Genes Is Associated with CpG Content

**DOI:** 10.1371/journal.pone.0043658

**Published:** 2012-08-27

**Authors:** Yuzuru Kato, Masami Nozaki

**Affiliations:** Department of Cell Biology, Research Institute for Microbial Diseases, Osaka University, Suita, Osaka, Japan; Massachusetts General Hospital, United States of America

## Abstract

In mammals, DNA methylation is restricted to cytosines of CpG dinucleotides, which are frequently found in short genomic regions including gene promoters. Methylation within CpG-rich regions around promoters tends to repress gene expression; thus, the CpG islands of housekeeping genes are normally unmethylated. We previously described a testis-specific single-exon gene containing a CpG-rich sequence that is methylated and thus repressed in somatic cells, whereas its expression in spermatogenic cells requires that it be hypomethylated. However, the relationship among the specific expression of spermatogenic genes, their methylation dynamics, and their CpG frequencies are poorly understood. Here, we analyzed the methylation patterns of the sphort genomic region around the transcription start site in spermatogenic cell-specific single-exon genes of various CpG contents. By using UniGene and Ensembl database analyses of the mouse genome and reverse transcription-PCR, we identified 39 single-exon genes that are exclusively expressed in spermatogeniccells. Regardless of their specific expression characteristics, genes containing higher (7 to 14 CpGs in 200 bp; mean = 12) and lower (2 to 6 CpGs in 200 bp; mean = 3.1) number ofCpG were hypo- and hyper-methylated, respectively, in all cell types examined, including spermatogeniccells. We found that genes with intermediate number of CpG (2 to 11 CpGs in 200 bp; mean = 6.9) are methylated in somatic cells, but not in male germ cells. These results suggest that DNA methylation dynamics of spermatogenic cell-specific single-exon genes are associated with CpG content, and the methylation status are stably maintained throughout male germ cell development.

## Introduction

In mammals, DNA methylation occurs at cytosine residues in CpG dinucleotides and is heritable during cell division. CpG cytosine methylation is thus an epigenetic marker that is essential for normal embryonic development [1]. This epigenetic modification of DNA is catalyzed by three DNA methyltransferases (Dnmts); Dnmt3a and Dnmt3b initiate *de novo* methylation and thereby establish new methylation patterns [2], and Dnmt1 is primarily involved in maintaining the methylation pattern [3]. In addition, another member of the Dnmt3 family, Dnmt3L, lacks the Dnmt catalytic domain and is specifically expressed during gametogenesis in the stages during which genomic imprints are established [4], [5].

Several studies have highlighted the importance of DNA methylation in male germ cells. Although mice with conventional null mutations of Dnmt3a die at approximately 3 weeks of age [2], conditional mutations of Dnmt3a targeted to the germ line result in a defect in spermatogenesis [6]. In the conditionally mutated germ cells, lack of methylation at imprinted genes and some repetitive sequences are observed [6], [7]. Male mice lacking Dnmt3L are infertile because they lack mature germ cells [4], [5] and display abnormal meiotic chromosome segregation [8], [9]. In these Dnmt3L-mutant mice, several repetitive and non-repetitive sequences, including imprint genes, in male germ cells are incompletely methylated [6]–[11]. These findings suggest that appropriate DNA methylation patterns are important for male germ-cell development.

The crucial role of DNA methylation in male germ cells is also supported by the presence of distinct methylation patterns. Some reports have shown that certain genome-wide DNA methylation patterns are unique to spermatozoa [11]–[13]. This unique state of DNA methylation might arise immediately after the genome-wide reprogramming event that occurs specifically in the primordial germ cells (PGCs) of the developing embryo between 10.5 and 12.5 days post-coitus (dpc) [14], when the DNA methylation patterns in imprinted and germ cell-specific genes are erased and reestablished [7], [15], [16]. Alternatively, *de novo* methylation and demethylation may occur in distinct CpG regions during spermatogenesis, as suggested by the observation that the methylation status of a few CpGs changes in spermatogenic cells [17].

Spermatogenesis is a highly programmed developmental process in which self-renewal and mitotic cell division in spermatogonia, meiotic cell division in spermatocytes, and spermiogenesis in spermatids act sequentially to produce spermatozoa. The temporal and spatial expressions of the specific genes that manage the organization of this complex process are strictly regulated.

Testis-specific expression of these genes is usually regulated by relatively short sequences in the gene promoter [18]–[20]. In addition to this type of regulatory mechanism involving the binding of transcription factors to cis-elements, we previously suggested that another type of mechanism, involving DNA methylation-induced gene suppression, might play a crucial role in gene expression during spermatogenesis. We found that the CpG-rich region in the spermatid-specific single-exon gene, *Tact1/Actl7b*, is methylated in somatic cells but not in spermatocytes and spermatids. Furthermore, transfection experiments with in vitro-methylated constructs indicated that methylation of *Tact1/Actl7b* represses its promoter activity in somatic cells [21]. These results revealed that germ cell-specific demethylation on the short genomic sequences near the promoter may be regulated for efficient transcription of the intronless gene. So far, several testis-sepcific single-exon genes have been found [22]–[25], but only limited analyses of their methylation status genes have been reported [26], [27].

In this study, we identified all of the single-exon genes that are expressed exclusively inspermatogenic cells and examined their methylation profiles around the promoter to explore the methylation dynamics of testis-specific genes during spermatogenesis. We found that these genes had a stable methylation status in the male germ line and that they could be sorted into three categories based on their methylation profiles: constitutively hypomethylated, constitutively hypermethylated, and germ line-specific hypomethylated genes. Furthermore, we found an association between their distinct methylation profiles and their CpG content. Genes with higher and lower number of CpG were constitutively hypo- and hypermethylated, respectively, and genes with intermediate number of CpG were hypomethylated in germ line cells and hypermethylated in somatic cells. These results suggest that the methylation status of spermatogenic cell-specific genes is regulated by their CpG content and that the hypomethylated status of the genes with intermediate CpG content might regulate their germ cell-specific expression.

## Results

### Identification of Spermatogenic Cell-specific Intronless Genes

To identify spermatogenic cell-specific intronless genes in the mouse genome, we first collected a dataset of testis-specific genes from the UniGene database (*Mus musculus*, UniGene build #159). We examined the structures of 246 testis-specific genes using GenBank and Ensembl database searches and found that 62 of them had open reading frames uninterrupted by introns, identifying them as candidate functional intronless genes (see Table S1). Of these genes, two (*1700084P21Rik* and *Tcp10a*) were not assembled at genome annotation status, and the genomic structures of nine (*1700012O15Rik*, *1700042G15Rik*, *1700066D14Rik*, *AA066038*, *4930571K23Rik*, *4933414I15Rik*, *1700110M21Rik*, *Cypt12*, and an unnamed gene) were not conclusively determined. The remaining 51 genes were taken as single-exon genes; 19 of these had been previously identified.

**Figure 1:**
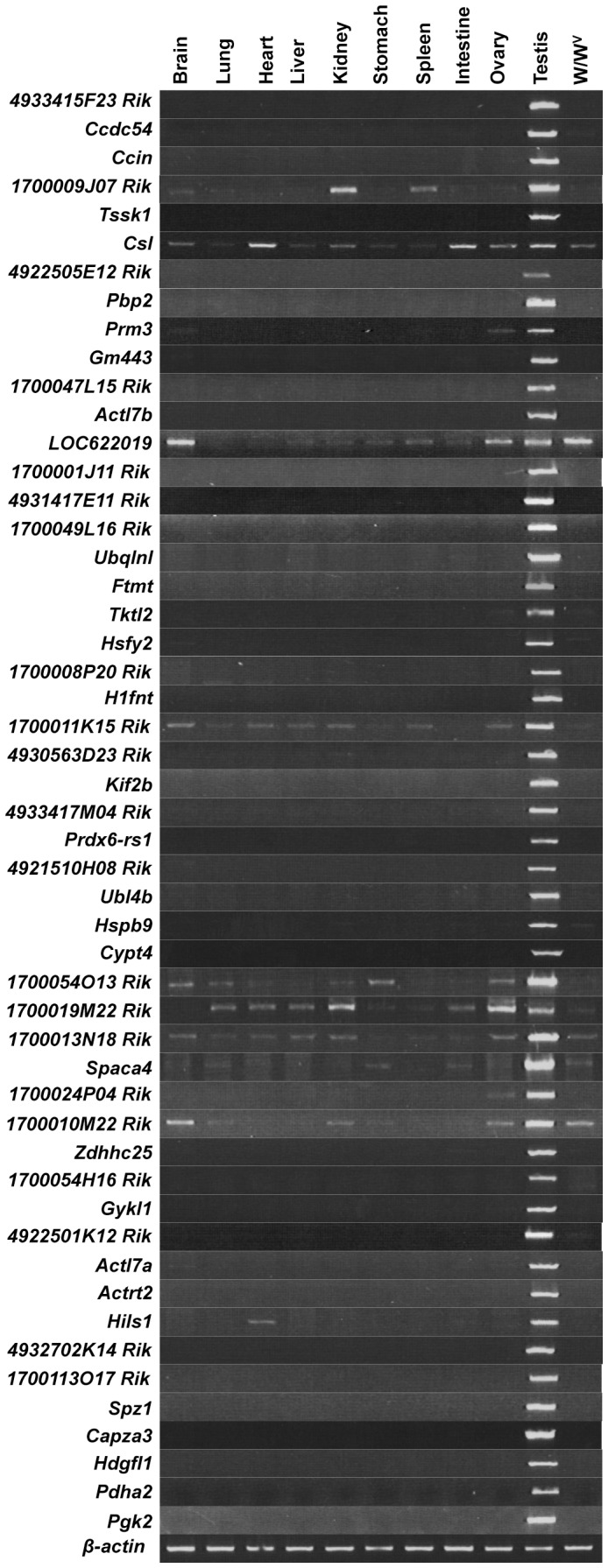
Spermatogenic cell-specific intronless genes are expressed in testis but not in any other tissues. Semiquantitative RT-PCR for the 51 intronless genes identified in Table S1 was carried out using total RNA isolated from adult mouse tissues. Thirty-nine genes were exclusively detected in testis and chosen for further study. The intensity of the β-actin band was used as a quantitative standard. The absence of genomic DNA in RNA samples is shown in Figure S1.

To confirm the expression of these intronless genes in the testis, we designed PCR primer sets and performed semiquantitative reverse transcription (RT)-PCR using total RNA prepared from various organs (Figure 1, Figure S1). In addition to testes from wild-type mice, we also used testes from *W/W^v^* mutant mice, which almost entirely lack germ cells as adults. Intense PCR signals were observed for all 51 genes in the testis, consistent with previous reports and expressed sequence-tag data for these genes in the UniGene database. Twelve of the genes (*1700009J07Rik*, *Csl*, *Prm3*, *LOC622019*, *1700011K15Rik*, *1700054O13Rik*, *1700019M22Rik*, *1700013N18Rik*, *Spaca4*, *1700024P04Rik*, *1700010M22Rik*, and *Hils1*) were also expressed in other tissues. The remaining 39 genes were expressed only in wild-type testis and were used as spermatogenic cell-specific intronless genes, although one of them, *Ftmt*, has been reported to be expressed in some other tissues [28].

### Characterization of Spermatogenic Cell-specific Intronless Genes

The intronless spermatogenic cell-specific genes identified in this study are shown in Table 1. A large number of intronless genes arise from retroposition of parental gene transcripts [29]. Since intronless genes are expected to be non-chromosomally-linked paralogs of their multiple exon-containing parental genes, we performed an Ensembl database analysis and homology search using BLASTP to find putative parental genes for the identified intronless genes. Paralogs were identified for 32 of the 39 genes (82%), suggesting that most spermatogenic cell-specific intronless genes are retrogenes generated from parental genes. However, we could not exclude the possibility that 10 of the genes (*Tssk1*, *Pbp2*, *Actl7a*, *Actl7b*, *Ubqlnl, Ftmt*, *Gykl1*, *Actrt2*, *4932702k14Rik*, and *1700113O17Rik*) might have arisen through segmental gene duplication from the other intronless paralogs. We were unable to find paralogs for the remaining seven intronless spermatogenic cell-specific genes, which might have diverged so much from their paralogs as to be unrecognizable by our methods.

**Table 1:** Identified spermatogenic cell-specific intronless genes.

Gene symbol	Chr	Ensemble ID	Paralogue			Orthologue	
			Gene symbol	Exon	Chr	Rat	Human
*4933415F23Rik*	1	ENSMUSG00000073730	*Ppp1r14b* (74%)	4	19	*RGD1309049* (78%)	
*Ccdc54*	16	ENSMUSG00000050685				*Ccdc54* (82%)	*CCDC54* (64%)
*Ccin*	4	ENSMUSG00000070999	*Klhl11* b (27%)	2	11	*Ccin* (99%)	*CCIN* (93%)
*Tssk1*	16	ENSMUSG00000041566	*Tssk5* b (38%)	8	15	*Tssk1* (96%)	*TSSK1B* (84%)
*4922505E12Rik*	1	ENSMUSG00000043429				*LOC289334* (80%)	*C1orf65* (55%)
*Pbp2*	6	ENSMUSG00000047104	*Pepb1* (79%)	4	5	*Pbp2* (88%)	
*Gm443*	5	ENSMUSG00000038044	*Cct8* (29%)	15	16	*MGC125233* (81%)	*CCT8L1* (61%)
*1700047L15Rik*	12						
Actl7b	4	ENSMUSG00000070980	Actin family (<39%)			*Actl7b* (85%)	*ACTL7B* (83%)
*1700001J11Rik*	9	ENSMUSG00000066639	*Rnf19a* b (84%)	10	15		
4931417E11Rik	6	ENSMUSG00000056197	*1200003C05Rik* (71%)	7	12	*RGD1562515* (91%)	
*1700049L16Rik*	10	ENSMUSG00000043859	Hn1l (88%)	5	17	*RGD1559903* (81%)	
*Ubqlnl*	7	ENSMUSG00000051437	*Ubqln1* b (36%)	11	13	*RGD1307202* (82%)	*UBQLNL* (45%)
*Ftmt*	18	ENSMUSG00000024510	*Fth1* (59%)	4	19	*Ftmt* (95%)	*FTMT* (67%)
Tktl2	8	ENSMUSG00000025519	*Tkt* (67%)	14	14	*Tktl2* (94%)	*TKTL2* (80%)
*Hsfy2*	1	ENSMUSG00000045336	*Hsf5* (13%)	6	11	*Hsfy1* b (95%)	
*1700008P20Rik*	7	ENSMUSG00000055826	*Tesc* (52%)	8	5	*RGD1311676* (83%)	
*H1fnt*	15	ENSMUSG00000048077				*H1fnt* (76%)	*H1FNT* (42%)
*4930563D23Rik*	16	ENSMUSG00000051728				*LOC498063* (86%)	
*Kif2b*	11	ENSMUSG00000046755	*Kif2a* (55%)	20	13	*Kif2b* (94%)	*KIF2B* (78%)
4933417M04Rik	1	ENSMUSG00000073479	*OTTMUSG00000005491* (37%)	2	11	*RGD1561646* (30%)	*FAM71A* (47%)
*Prdx6-rs1*	2	ENSMUSG00000050114	*Prdx6* (86%)	5	1		Pseudo
*4921510H08Rik*	10	ENSMUSG00000047025				*LOC500825* (91%)	*C12orf12* (62%)
*Ubl4b*	3	ENSMUSG00000055891	*Ubl4* (34%)	4	X	*LOC684560* b (96%)	*UBL4B* (62%)
*Hspb9*	11	ENSMUSG00000017832	*Hspb8* b (27%)	3	5	*Hspb9* (76%)	*HSPB9* (54%)
*Cypt4*	9	ENSMUSG00000047995	*Cypt3* (80%)	2	X		
*Zdhhc25*	15	ENSMUSG00000054117	*Zdhhc7* b (41%)	8	8	*LOC300323* b (83%)	
*1700054H16Rik*	11		*Pom121* b (28%)	13	5		*DKFZp564* b (42%)
*Gykl1*	18	ENSMUSG00000053624	*Gyk* (82%)	21	X	*Gk-rs1* (93%)	
*4922501K12Rik*	14	ENSMUSG00000078127	*Gm93* (17%)	4	8	*4922501K12Rik* (67%)	*C10orf73* (47%)
*Actl7a*	4	ENSMUSG00000070979	Actin family (<37%)			*Actl7a* (96%)	*ACTL7A* (85%)
*Actrt2*	4	ENSMUSG00000051276	Actin family (<49%)			*Actrt2* (95%)	*ACTRT2* (80%)
*4932702K14Rik*	17	ENSMUSG00000046173	*Pabpc1* (80%)	15	15	*Pabpc3* (94%)	
*1700113O17Rik*	2	ENSMUSG00000062651	H2A histone family (<39%)				
*Spz1*	13	ENSMUSG00000046957				*Spz1* (76%)	*SPZ1* (48%)
*Capza3*	6	ENSMUSG00000041791	*Capza2* (35%)	10	6	*Capza3* (97%)	*CAPZA3* (91%)
*Hdgfl1*	13	ENSMUSG00000045835	*Hdgf* (33%)	6	3	*Pwwp1* (68%)	*HDGFL1* (36%)
*Pdha2*	3	ENSMUSG00000047674	*Pdha1* (75%)	11	X	*Pdha2* (92%)	*PDHA2* (74%)
*Pgk2*	17	ENSMUSG00000031233	*Pgk1* (83%)	11	X	*Pgk2* (90%)	*PGK2* (85%)

A Putative paralogs and orthologs were basically identified based on Ensemble database search.

b BLASTP was carried out to identify paralogs and orthologs in the case that paralogs and orthologs were not registered in Ensemble database.

The most identical gene was represented when several candidates were registered in the database. Paralogs in a gene family were represented by their family name. Percentages indicate identities of amino acid sequence for each intronless gene.

Analysis of the chromosomal locations of the 32 retroposed gene pairs revealed an apparently random insertion of the retrogenes into almost all chromosomes except chromosomes 12 and 19 and the sex chromosomes. In contrast, a disproportionately large number of putative parental genes were found on the X chromosome. On the basis of the assumption that the number of parental genes on calculated that the mouse X chromosome has generated a 4-fold excess of spermatogenic cell-specific retrogenes, consistent with a previous report [30].

We also examined orthologs of the intronless spermatogenic cell-specific genes in human and rat. Of the 39 mouse genes, we found that 23 (59%) had orthologs in both the human and rat genomes (Table 1), suggesting that these genes originated before the divergence of the primate and rodent lineages. Of these orthologous genes, two (*Pdha2* and *Pgk2*) had high levels of deduced amino acid identity with both their paralogs and their orthologs, implying that they have functions similar to those of their paralogs. For six genes (*Ccin*, *Tssk1*, *Actl7a*, *Actl7b*, *Actrt2*, and *Capza3*) the levels of identity were markedly higher for their orthologs than their paralogs, suggesting that they had acquired divergent functions. Four of the genes (*1700047L15Rik*, *1700001J11Rik*, *Cypt4*, and *1700113O17Rik*) had no orthologs in rat or human, suggesting that they are mouse-specific. However, it is unlikely that these four genes were generated after the divergence of mouse from rat, since their levels of deduced amino acid identity with their paralogs are low.

### Timing of Intronless Gene Expression during Spermatogenesis

To investigate the expression profiles of the spermatogenic cell-specific intronless genes during spermatogenesis, we performed RT-PCR analysis using total RNA isolated from testes of 7-, 14-, 21-, and 35-day-old mice (D7, D14, D21, and D35 mice, respectively). The first spermatogenic cycle initiates a few days after birth and proceeds during postnatal development; consequently, the juvenile mouse testis at each of these days contains a different subset of spermatogenic cell types. Type A and B spermatogonia are present on D7. On D14, meiotic prophase is initiated, and the germ cells become pachytene spermatocytes. Haploid spermatids appear in increasing numbers on D21, and all stages of spermatogenic cells, including spermatozoa, appear on D35 [31].

**Figure 2:**
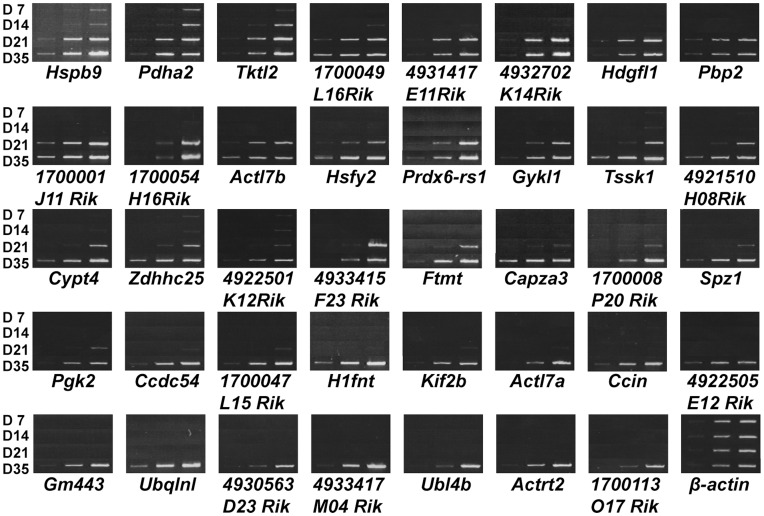
Expression levels of 39 spermatogenic cell-specific intronless genes are increased in later stages of spermatogenesis. Semiquantitative RT-PCR was carried out using total RNA isolated from D7, D14, D21, and D35 testes. The number of PCR cycles increases from left to right in each panel, as described in Table S2. The β-actin band represents equal expression levels for each stage. The absence of genomic DNA in RNA samples is shown in Figure S2.

As shown in Figure 2 (see also Figure S2), expression of all the genes was upregulated during the course of testis development. Signals for *Hspb9* and *Pdha2* were detected in D7 and D14 testes and were higher in D21 and D35 testes. Expression of *Tktl2* was observed at D14 and was higher at D21 and D35. For most of the genes, strong signals of varying intensities were detected on D21, indicating expression at the late spermatocyte stage. The remaining genes were not detected until D35, indicating expression exclusive to the round spermatid stage. The observed expression profiles were consistent with previous reports [32]–[39] and conclusively indicated that spermatogenic cell-specific intronless genes have a strong bias for expression during the late meiotic spermatocyte to haploid spermatid stages.

### Methylation Profile of Intronless Genes during Spermatogenesis

We previously demonstrated that DNA methylation of the CpG-rich region in the *Tact1/Actl7b* gene represses its expression in somatic cells and that demethylation is necessary for its expression in spermatogenic cells [21]. In another study, gradual loss of methylation was shown to occur in the *Pgk2* gene during male germ cell development [27]. Moreover, several studies showed that the expression of the testis-specific genes were usually regulated by short genomic region including the promoter [18]–[20]. These observations prompted us to investigate whether demethylation is a general requirement for expression of spermatogenic cell-specific intronless genes. To address this issue, we carried out combined bisulfite restriction analysis (COBRA) of 20 selected intronless genes using genomic DNA from D7, D14, D21, and D35 mouse testis and from adult mouse liver. Nineteen of these genes contained restriction enzyme sites within 100 bp of the transcription start sites (Figure S3). The 20th gene, *Actl7b*, had a restriction site for COBRA at position +340; its methylation status is known to be coordinately regulated with two CpG sites within 100 bp of the transcription start site [21].

**Figure 3:**
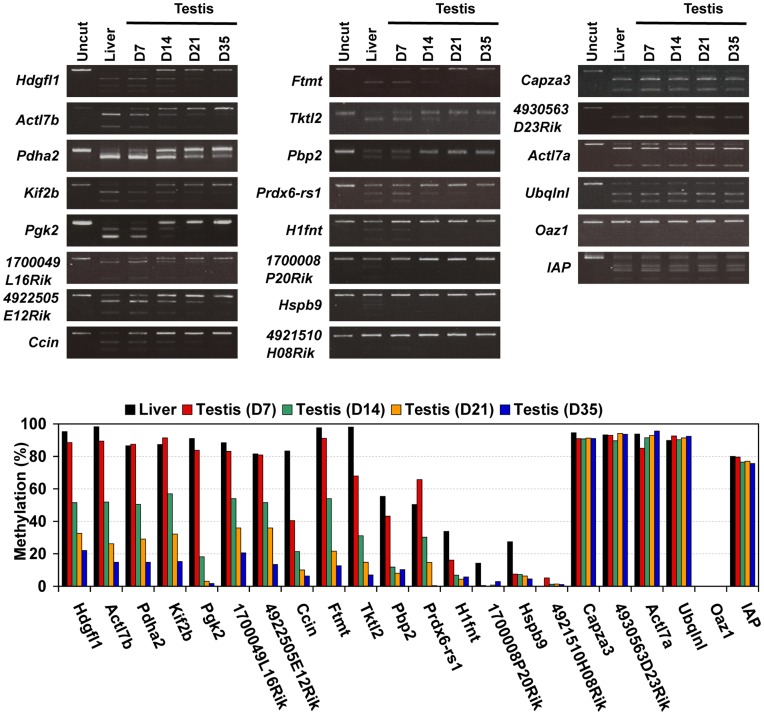
Methylation profiles of spermatogenic cell-specific intronless genes are classified three groups in juvenile mouse testis. COBRA was carried out to examine the methylation status of the selected genes, the CpG island in the *Oaz1* gene, and constitutively methylated endogenous IAP retroviruses in genomic DNA isolated from D7, D14, D21, and D35 testes and adult liver. Sodium bisulfite-treated DNA was amplified with specific primers (details in Table S3) and digested with HpyCH4 IV (recognition site: ACGT), Taq I (TCGA) or Acc II (CGCG).

The twenty genes were sorted into three groups based on their observed methylation patterns in D7, D14, D21, and D35 testis and adult liver (Figure 3). The first group comprised four genes (*H1fnt*, *1700008P20Rik*, *Hspb9*, and *4921510H08Rik*) that were hypomethylated in all samples. Their low methylation level essentially resembled that of *Oaz1*, which has a COBRA restriction site within a CpG island, although *H1fnt*, *1700008P20Rik*, and *Hspb9* were moderately methylated in liver. In contrast, the four genes of the second group (*Capza3*, *4930563D23Rik*, *Actl7a*, and *Ubqlnl*) were hypermethylated in all tissues examined, similar to endogenous IAP retroviruses, which are known to be highly methylated in both somatic tissues and testis [40]. The methylation statuses of the eight genes in the first and second groups were essentially stable in spermatogenic cells and somatic cells.

**Figure 4:**
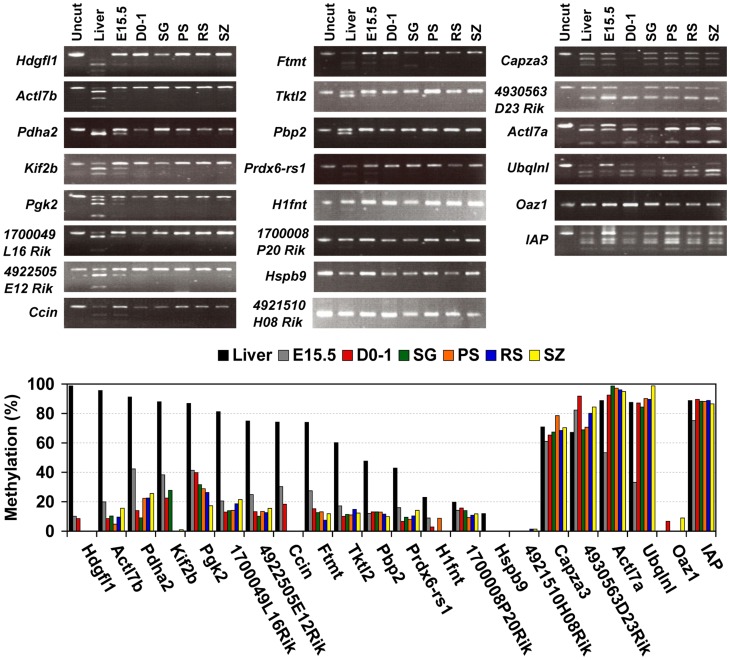
Methylation profiles of spermatogenic cell-specific intronless genes are classified three groups in male germ cells. COBRA was carried out to examine the methylation status of the selected genes, the CpG island in the Oaz1 gene, and constitutively methylated IAP in genomic DNA isolated from 15.5 dpc embryonic (E15.5) and neonatal (D0–1) gonocytes, spermatogonia (SG), pachytene spermatocytes (PS), round spermatids (RS), epididymal spermatozoa (SZ), and adult liver. The sodium bisulfite reaction and restriction enzyme digestion were carried out as described in Figure 3. The COBRA results are also represented by bar graphs.

The remaining 12 genes (*Hdgfl1*, *Actl7b*, *Pdha2*, *Kif2b*, *Pgk2*, *1700049L16Rik*, *4922505E12Rik*, *Ccin*, *Ftmt*, *Tktl2*, *Pbp2*, and *Prdx6-rs1*), which formed the third group, were methylated at high levels in liver and D7 testis. The methylation levels of these genes gradually decreased in accordance with testis maturation, during which time the fraction of germ cells in the testis cell population increases [31] and spermatogenesis proceeds. To determine whether methylation levels of these genes were constitutively low in germ cells or if they decreased during spermatogenesis, we examined the methylation status of these genes in spermatogenic cells at each stage, including spermatogonia, pachytene spermatocytes, round spermatids, and spermatozoa. We found that they were highly methylated in liver but hypomethylated or nonmethylated in spermatogenic cells (Figure 4). Furthermore, their methylation levels were also low in pre-spermatogonia, including embryonic and neonatal gonocytes, suggesting that they were hypomethylated in a germ cell-specific manner. In contrast, methylation levels of the genes in the first and second groups were similar in isolated germ cells and in testis, although *Actl7a* and *Ubqlnl* methylation levels might have decreased transiently in embryonic germ cells.

### Distinct Methylation Profiles Correlate with CpG Content

**Figure 5:**
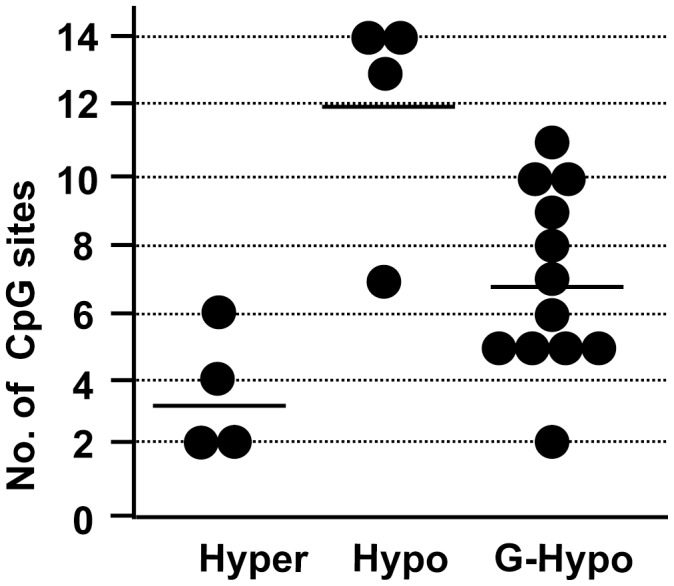
Methylation levels of the three categorized genes depend on their CpG contents. The number of CpG sites within a 200-bp segment containing the COBRA restriction sites in the 20 intronless genes described in Figures 3 and 4 was determined, and the results plotted as a histogram. Each dot indicates a gene in each group classified according to the methylation profiles shown in Figures 3 and 4. Hyper, constitutive hypermethylated; Hypo, constitutive hypomethylated; G-Hypo, germ cell-specific hypomethylated.

The presence of three distinct types of methylation profiles among the spermatogenic cell-specific intronless genes implies that the genes in these groups interact differently with the regulatory machinery. Methylation level has been shown to be related to the number of CpG dinucleotides within distinct genome regions [41]. To gain insight into the variety of methylation profiles, we determined the number of CpG dinucleotides in a 200-bp segment containing the COBRA restriction sites. As shown in Figure 5, the frequency of CpG dinucleotides in the genes differed among the three groups. Genes belonging to the constitutively hypermethylated group had low CpG content; in 200 bp, they had 2 to 6 CpGs (mean = 3.1). Genes of the constitutively hypomethylated group had high CpG content; in 200 bp, there were 7 to 14 CpGs (mean = 12). These results suggest that genomic regions with lower CpG number are stably methylated in any tissue, whereas those with higher CpG number tend to maintain a hypomethylated state. The genes that were demethylated in male germ cells but methylated in somatic cells had intermediate CpG number (2–11 CpGs in 200 bp; mean = 6.9). The range of CpG content in this intermediate group included genes of low CpG frequency (<6 in 200 bp) but no genes of very high frequency (>12 in 200 bp).

## Discussion

### Spermatogenic Cell-specific Intronless Genes

In our analysis of the mouse UniGene database (UniGene build #159), we identified 246 testis-specific genes. To select the primary hits from the database searches, we restricted the number of expressed sequence-tag entries to at least 10. Using RT-PCR, we determined that 39 of these intronless genes were expressed in spermatogenic cells. Of these genes, 36 (92%) were expressed in mice older than 21 days, suggesting that most testis-specific genes selected in this analysis were expressed during the late-meiotic and post-meiotic stages of spermatogenesis.

Previous UniGene searches yielded 24 and 28 uncharacterized genes specifically expressed in spermatocytes and round spermatids, respectively [42], [43]. Our list included 14 (58.3%) of the spermatocyte-specific genes and 8 (28.6%) of the spermatid-specific genes. Of the remaining 28 previously identified genes, 26 were not included in our list because they had either been eliminated from the most recent version of the database (9 genes), had entries in various tissues other than testis (10 genes), or had a low number of entries (9 genes). Although the remaining two genes (*Mm. 45611* and *437189*) satisfied our criteria, our analysis did not select them, for unknown reasons.

One-fourth (62/246) of all the testis-specific genes we identified consisted of a single exon. In contrast, only 5.6% (2/36) of spermatogonially expressed germ cell-specific genes are intronless [44]. Therefore, a lack of introns may be characteristic of genes expressed during later stages of spermatogenesis. In addition, a large percentage of testis-specific intronless genes are probably retroposons (32/39, 82%), indicating that retrogenes tend to be expressed at later stages of spermatogenesis.

Retrogenes generally give rise to pseudogenes; as inserted cDNA is reverse-transcribed from mRNA, it lacks a genuine promoter sequence and, therefore, cannot be expressed in any tissue. Thus, our results suggest that the late stage of spermatogenesis provides an appropriate environment for retroposon transcription and facilitates gene expression from promoter-like sequences. Furthermore, these genes might have acquired their high level of transcriptional activity in pachytene spermatocytes or round spermatids by accumulating favorable mutations in their promoters during evolution. Otherwise, this stage of germ cells might possess an accessible environment for retroposition. It is possible that the retrogenes were simply inserted into chromatin regions that are transcription-prone.

### Regulation of Methylation Status

We classified the identified spermatogenic cell-specific intronless genes into three distinct groups on the basis of their DNA methylation profiles, which were associated with their CpG content. The genes with lower CpG number were constitutively hypermethylated in somatic and germ cells. In contrast, genes with higher CpG number were hypomethylated in both somatic and germ cells. These two groups accounted for 40% of the spermatogenic cell-specific intronless genes.

We also demonstrated that the remaining intronless genes those with intermediate CpG number were hypomethylated in spermatogenic cells and hypermethylated in somatic cells. These observations agree with the reported promoter methylome analysis in which methylated DNA from human primary fibroblasts and sperm was immunoprecipitated and analyzed using microarrays [41]. Other studies have shown that the haploid-specific genes with lower CpG number *Prm1*, *Prm2*, and *Tnp2*, which have two exons are hypermethylated throughout spermatogenesis [45], [46], and another haploid-specific, two-exon gene with intermediate CpG number, *Tnp1*, is hypomethylated during spermatogenesis [46]. Taken together, these analyses suggest that a correlation between methylation status and CpG content is a common feature of the mammalian genome. We also found that the intronless genes with intermediate CpG number were hypomethylated in gonocytes in neonatal males and in male embryos at 15.5 dpc, indicating that these genes are hypomethylated throughout the male germ line.

Dynamic changes in DNA methylation occur during PGC development in the genital ridge and in early embryogenesis. The differentially methylated regions of some imprinted genes and some repetitive sequences are demethylated in PGC in the post-migrating stage but not in somatic cells [15], [16]. These observations suggest that genome-wide demethylation occurs in germ cells at these stages, during which testis-specific hypomethylated intronless genes may also be demethylated. The hypomethylated status of the genes and PGC expression of the *Mvh* gene [47] are maintained during spermatogenesis until spermatozoa form, although the differential methylation regions of paternally imprinted genes are remethylated after 14.5 dpc [7].

Dnmt3L, an activator of Dnmt3a and Dnmt3b [48]–[52], is indispensable for methylation of imprinted genes and repetitive sequences in gonocytes [7], indicating that Dnmt3L/Dnmt3a and Dnmt3L/Dnmt3b complexes can target these genome regions but not testis-specific hypomethylated intronless genes. It was also reported that methylation of histone H3 Lysine 4 blocks the association of Dnmt3L to the chromatin [53], suggesting that testis-specific hypomethylated intronless genes were marked with the histone modification preceded to the d*e novo* methylation. In addition, genome-wide remethylation occurs during early embryogenesis. Although intronless genes with higher CpG number, are hypomethylated in all cell types, testis-specific hypomethylated intronless genes are hypermethylated in somatic cells, indicating that genome sequences of the genes might be a determinant of *de novo* methylation during development, as mentioned previously [41].

### Relationship between DNA Methylation and Gene Expression

DNA methylation is thought to affect transcriptional silencing by two mechanisms. First, methylated CpGs can directly inhibit the association of some transcriptional factors with their regulatory sequences [54]; second, proteins that recognize methyl-CpG can elicit the repressive potential of methylated DNA [55], [56]. In the latter mechanism, methyl-CpG-binding proteins bound to methylated CpGs are thought to recruit transcriptional co-repressors to modify the surrounding chromatin, creating a gene-silencing environment [57]–[62]. In the present study, however, we showed that intronless genes with lower CpG number are expressed in round spermatids, in which they have hypermethylated CpGs. It appears that a high density of methylated CpGs is required for methylation-dependent gene silencing. Alternatively, the positions of the methylated CpGs might not be appropriate for gene silencing.

Some promoters of testis-specific genes can activate transcription in cultured somatic cells [21], [63], [64]. We previously demonstrated that methylation of the testis-specific *Tact1/Actl7b* gene with intermediate number of CpG dramatically reduced its expression relative to an unmethylated control in a transient transfection assay [21]. More than 40 cancer/testis antigens have been identified; this class of tumor antigen is encoded by genes that are normally expressed in testis germ cells but not in normal somatic tissues [65], and promoter demethylation appears to be a key element in their expression. The cancer/testis antigen genes are methylated in normal somatic tissues and are activated by demethylation during spermatogenesis. The global hypomethylation that occurs during carcinogenesis is associated with their expression [66], [67]. These observations suggest that DNA methylation of testis-secific hypomethylated genes in somatic cells play a role in gene silencing.

However, certain mechanisms other than DNA methylation might be involved in repression of testis-specific hypomethylated intronless genes in male germ cells, despite their hypomethylated states. Since a crucial epigenetic mark of transcriptional silencing is the methylation of histone H3 Lysine 9 (H3K9) by the histone lysine methyltransferase G9a [68], [69], this methylation might be involved in the repression the genes. The genome-wide pattern of dimethylated H3K9 is established in pre-leptotene spermatocytes by G9a and is erased in pachytene spermatocytes by the mono/dimethylated H3K9-specific demethylase JHDM2A [70]. In addition, in one study, H3K9 methylation was correlated with *Tnp1* and *Prm1* gene silencing in the round spermatids of mutant mice with a disrupted *JHDM2A* gene [71]. On the other hand, in another study, dimethylated H3K9 was not enriched in the proximity of the Prm1 gene promoter in spermatogenic cells at any stage [72]. These inconsistent data show that the exact nature of the link between histone modifications and silencing of testis-specific hypomethylated genes in male germ cells remains to be elucidated.

## Materials and Methods

### Tissue Preparation and Germ Cell Isolation

All tissues and cells were isolated from Slc: ICR mice (Japan SLC, Inc.). TRIzol reagent (Invitrogen) was used to isolated total RNA from adult mouse tissues, including brain, lung, heart, liver, kidney, stomach, spleen, ovary, wild-type testis, and *W/W^v^* testis, and from testes of D7, D14, D21, and D35 mice.

Gonocytes and spermatogonia were isolated by FACS. Testes from 15.5 dpc embryos and from neonates at day 0–1 and 7 were dissociated with 0.1% trypsin (Nacalai Tesque) at 32°C for 10 min. The trypsin reaction was stopped by the addition of DMEM containing 10% FBS and passed through nylon mesh. The dissociated testis cells were incubated with 10% FBS/DMEM at 32°C for 15 min and then washed three times with ice-cold phosphate-buffered saline containing 0.1% bovine serum albumin and 0.01% sodium azide.

The isolated testis cells were incubated with anti-EpCAM antibody (Santa Cruz Biotechnology) for 60 min and then with Alexa Fluor 488-conjugated goat anti-rat IgG (Molecular Probes) and used for FACS analysis (FACS Aria, BD Biosciences). The purity of the germ cells was determined by immumostaining using anti-GCNA1 antibody and Alexa Fluor 594-conjugated goat anti-rat IgM (Molecular Probes). Approximately 80% of the cells exhibited signals for both EpCAM and GCNA1.

Pachytene spermatocytes and round spermatids were also isolated using FACS. The tunica albuginea was removed from adult testis, and the seminiferous tubules were dissociated using sequential enzymatic digestion by collagenase (100 U/ml, Worthington) at 32°C for 15 min and then by 0.1% trypsin at 32°C for 15 min. Five minutes into the trypsin treatment, DNase I (2 µg/ml; Sigma) was added. The trypsin reaction was stopped by the addition of trypsin inhibitor (0.1%; Nacalai Tesque), and the resulting suspension was passed through nylon mesh. The cells were then incubated with PBS containing 1% FBS, 1 µg/ml Hoechst 33342, and 2 µg/ml propidium iodide (Invitrogen) at 32°C for 20 min. FACS Aria was performed with a 407-nm violet laser. The pachytene spermatocytes and round spermatids were successfully separated by their DNA content and collected [73]. The purity of the cell preparations was evaluated by immunostaining of SCP3 for pachytene spermatocytes (75%) and by examination of cell morphology for round spermatids (90%). Epididymal spermatozoa were collected using a standard protocol.

All animal experiments were approved by the Animal Care and Use Committee of the Research Institute for Microbial Diseases, Osaka University.

### Reverse Transcription (RT)-PCR

One microgram of total RNA was treated with DNase I at 37°C for 20 min, and the cDNA was reverse-transcribed using SuperScript III (Invitrogen) at 50°C for 60 min. Ex Taq Polymerase (TaKaRa) was then used for PCR with the primers and conditions shown in Table S2.

### Combined Bisulfite Restriction Analysis (COBRA)

One microgram of genomic DNA was treated with bisulfite using an EZ DNA methylation Kit (Zymo Research), and 50 ng of the bisulfite-treated DNA was used for PCR with the primers and conditions shown in Table S3. The PCR products were ethanol-precipitated and digested with HpyCH4 IV (NEB) and AccII (TaKaRa) at 37°C and with TaqI (TaKaRa) at 60°C for 1 h. The digested PCR products were separated by 1.6%-agarose gel electrophoresis. Signal intensities were measured using an Image Reader FLA-7000 (Fujifilm).

## Supporting Information

Figure S1 **The absence of genomic DNA in 51 RNA samples.** RT-PCRs were performed using RNA samples with or without reverse transcriptase reaction.
(TIF)

Figure S2 **The absence of genomic DNA in RNA samples.** RT-PCRs were performed as in Figure S1.
(TIF)

Figure S3 **Schematic representation of CpG sites of 20 selected spermatogenic cell-specific intronless genes.** CpG sites within 200 bp of the transcription start site are shown with vertical lines. A dotted arrow indicates the transcription start sites. Restriction enzyme sites are indicated by capital letters. A, AccII; H, HpyCH4 IV; T, TaqI.
(TIF)

Table S1 **Testis-specific genes in the mouse.**
(DOC)

Table S2 **Primer sequence and parameters for RT-PCR.**
(DOC)

Table S3 **Primer sequence and parameters for bisulfite PCR.**
(DOC)
