# Supplementary figures and images for: Distinct DNA Methylation Dynamics of Spermatogenic Cell-Specific Intronless Genes Is Associated with CpG Content
Source: PLoS One. 2012 Aug 27;7(8):e43658. doi: 10.1371/journal.pone.0043658 (PMC3428356; doi:10.1371/journal.pone.0043658)

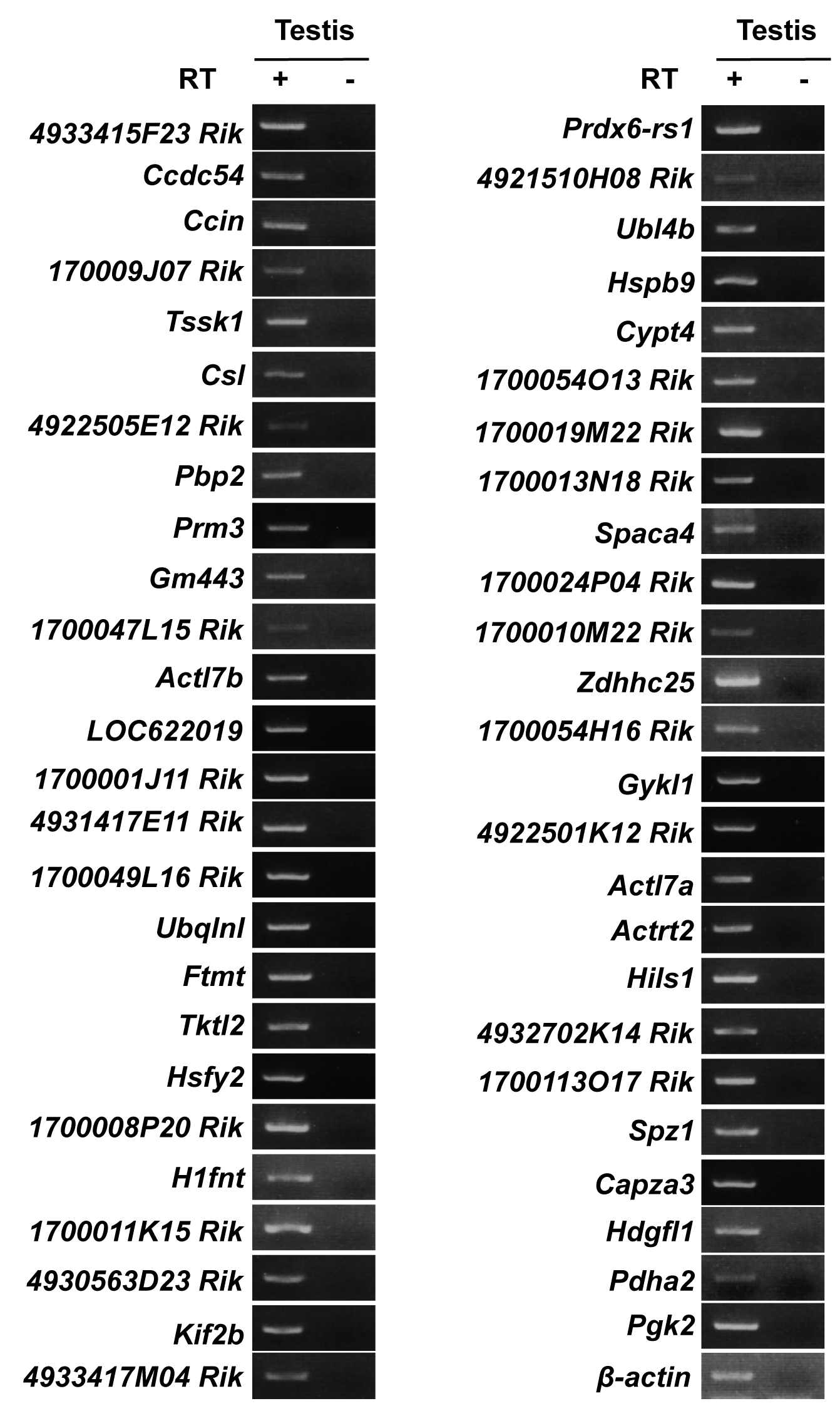

Supplement: Figure S1 — The absence of genomic DNA in 51 RNA samples. RT-PCRs were performed using RNA samples with or without reverse transcriptase reaction. (TIF) [file pone.0043658.s001.tif]

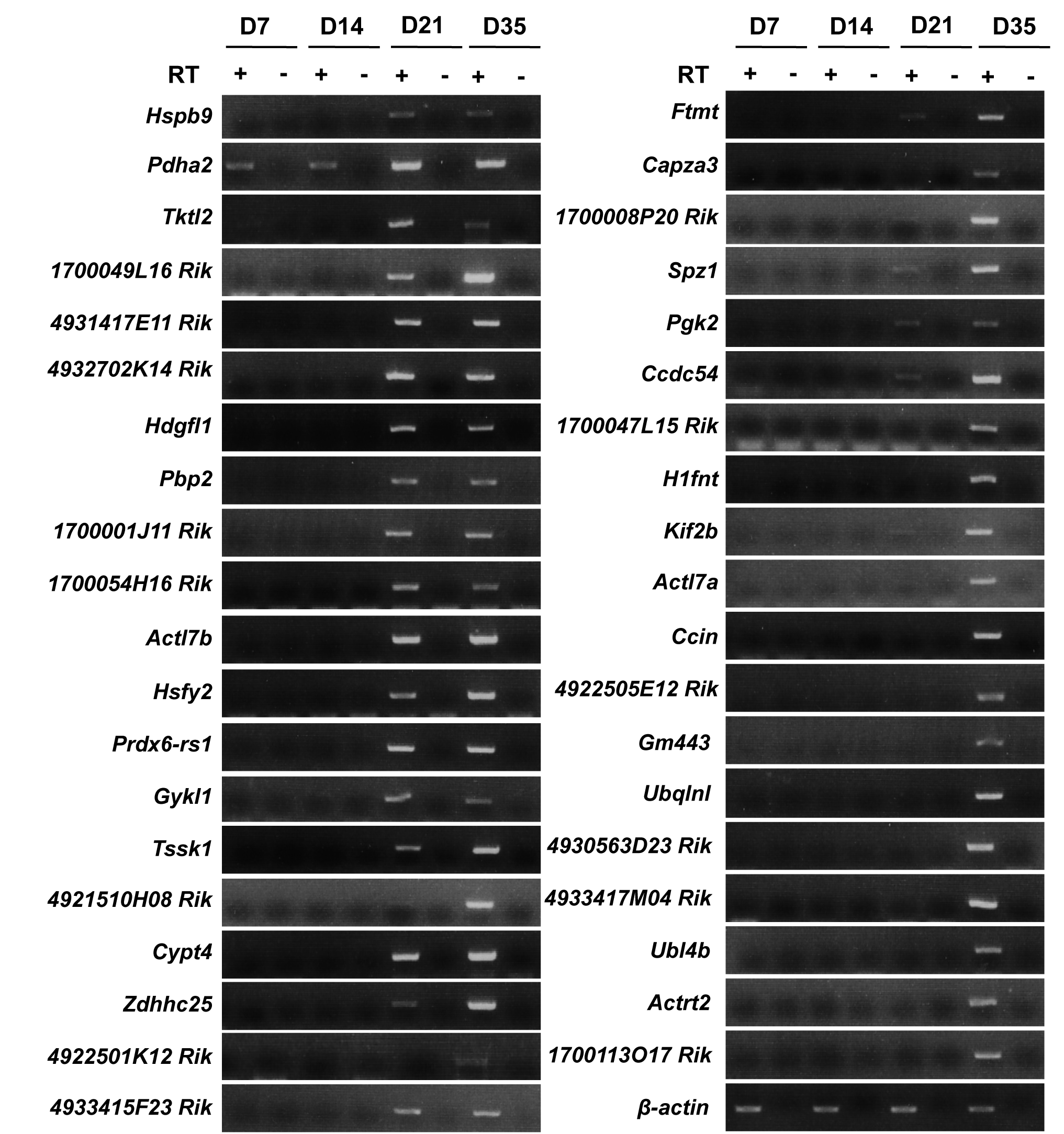

Supplement: Figure S2 — The absence of genomic DNA in RNA samples. RT-PCRs were performed as in Figure S1. (TIF) [file pone.0043658.s002.tif]

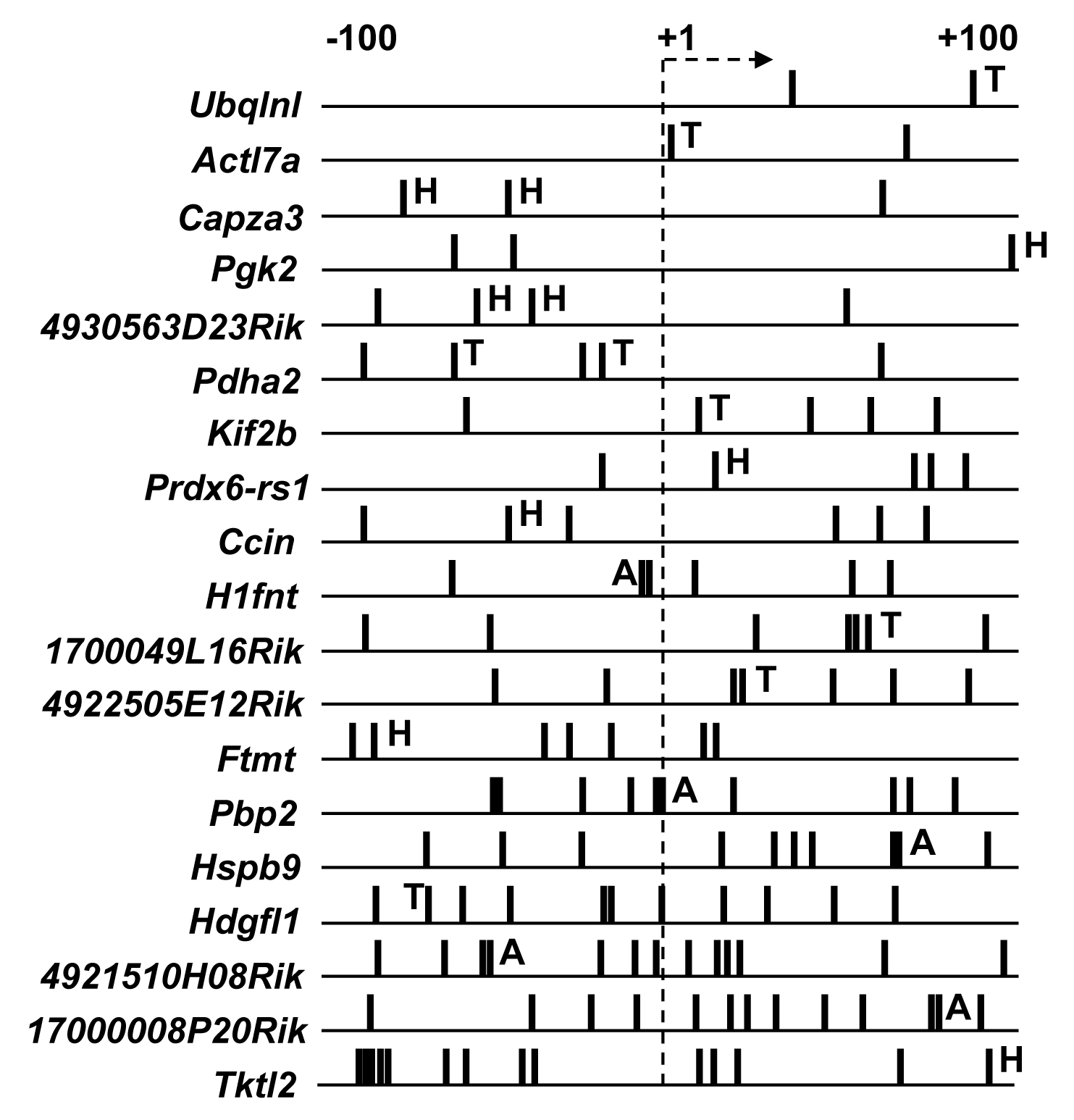

Supplement: Figure S3 — Schematic representation of CpG sites of 20 selected spermatogenic cell-specific intronless genes. CpG sites within 200 bp of the transcription start site are shown with vertical lines. A dotted arrow indicates the transcription start sites. Restriction enzyme sites are indicated by capital letters. A, AccII; H, HpyCH4 IV; T, TaqI. (TIF) [file pone.0043658.s003.tif]
