# Supplementary material for: Distinct DNA Methylation Dynamics of Spermatogenic Cell-Specific Intronless Genes Is Associated with CpG Content
Source: PLoS One. 2012 Aug 27;7(8):e43658. doi: 10.1371/journal.pone.0043658 (PMC3428356; doi:10.1371/journal.pone.0043658)
Supplement: Table S1 — Testis-specific genes in the mouse. (DOC) [file pone.0043658.s004.doc]

**Table S1**. Testis-specific genes in the mouse

| **Mm. No.^a^** | **Gene Symbol** | **Gene ID** | **Exon^b^** | **Chr** |
| --- | --- | --- | --- | --- |
| 431297 | 4933415F23Rik | 66755 | 1 | 1 |
| 425989 | *Tcp10b* | 21461 | 12 | 17 |
| 425445 | *1700039E15Rik* | 76713 | 6 | 7 |
| 425335 | *1700013D24Rik* | 76921 | 3 | 6 |
| 425115 | *1700030G11Rik* | 70065 | 4 | 2 |
| 425024 | *Tnp1* | 21958 | 2 | 1 |
| 424983 | *1700125D06Rik* | 68233 | 3 | 4 |
| 422869 | *Ccdc54* | 69339 | 1 | 16 |
| 398302 | *1700109G14Rik* | 67355 | (3<) | 14 |
| 396211 | *D19Ertd386e* | 52013 | 6 | 19 |
| 395491 | *1700012O15Rik* | 75488 | (1) | 2 |
| 394667 | *1700026D11Rik* | 70371 | (4<) | 2 |
| 393774 | *1700042G15Rik* | 73349 | (1) | 4 |
| 392746 | *Frs3* | 107971 | 7 | 17 |
| 392402 | *1700066D14Rik* | 67925 | (1) | 10 |
| 391543 | *1700034J05Rik* | 73344 | 3 | 6 |
| 391200 | *1700017D01Rik* | 69369 | (7<) | 19 |
| 390750 | *1700049K14Rik* | 73382 | 5 | 14 |
| 390691 | *Nkx2-6* | 18092 | 2 | 14 |
| 390629 | *1700029F12Rik* | 66479 | 3 | 13 |
| 389722 | *4930449I24Rik* | 67410 | 5 | 5 |
| 387526 | *AA066038* | 53960 | (1) | 16 |
| 386790 | *1700052I22Rik* | 67340 | (2<) | 12 |
| 383210 | *Tsga8* | 60600 | 2 | X |
| 379326 | *Xmr* | 382277 | 9 or 6 | X |
| 379188 | *Rnf36/Trim69* | 70928 | 6 | 2 |
| 371643 | *1700008I05Rik/Tcp11* | 71841 | 9 | X |
| 358754 | *1700084P21Rik* | 73498 | 1 | 14 |
| 349600 | *4930571K23Rik* | 75861 | (1) | 7 |
| 348215 | *Ccin* | 442829 | 1 | 4 |
| 348012 | *1700009J07Rik* | 75188 | 1 | 10 |
| 347554 | *Tssk1* | 22114 | 1 | 16 |
| 341880 | *EG627607* | 627607 | 14 | 12 |
| 339192 | *Csl* | 71832 | 1 | 10 |
| 338103 | *4922505E12Rik* | 433386 | 1 | 1 |
| 335525 | *Slc22a14* | 382113 | 12 | 9 |
| 335431 | *Iqcf4* | 67320 | 3 | 9 |
| 334503 | *Adam39* | 546055 | 2* | 8 |
| 334092 | *Ubqln3* | 244178 | 2* | 7 |
| 333542 | *4930401F20Rik* | 243822 | 7 | 7 |
| 333010 | *1700074P13Rik* | 73481 | 7 | 6 |
| 332809 | *LOC640324* | 640324 | 2 | 5 |
| 332291 | *1700001C02Rik* | 75434 | 4 | 5 |
| 329435 | *Gm93* | 225497 | 4 | 18 |
| 329132 | *Arrdc5* | 76920 | 3 | 17 |
| 326730 | *LOC382133* | 382133 | 2* | Y |
| 325769 | *Prm2* | 19119 | 2 | 16 |
| 308822 | *4933422H20Rik* | 432613 | 8 | 11 |
| 307436 | *Als2cr11* | 73463 | 13 | 1 |
| 307084 | *1700019A02Rik* | 69397 | 8 | 1 |
| 296649 | *Gm172/Ccdc110* | 212392 | 7 | 8 |
| 293026 | *BC061237* | 385138 | 5 | 14 |
| 293018 | *Pbp2* | 76400 | 1 | 6 |
| 292326 | *1700029M20Rik* | 73937 | 2 | 4 |
| 291887 | *Prm3* | 19120 | 1 | 16 |
| 291732 | *1700003E24Rik* | 100038763 | 2 | X |
| 291521 | *Gm443* | 242891 | 1 | 5 |
| 288704 | *1700007I06Rik* | 69306 | 4 | 11 |
| 279725 | *1700015G11Rik* | 75529 | 3 or 4 | 7 |
| 276558 | *1700047L15Rik* | 73309 | 1 | 12 |
| 275677 | *Lyzl6* | 69444 | 5 | 11 |
| 273326 | *1700011F03Rik* | 74221 | 4 | 6 |
| 273301 | 1700023D19Rik | 75510 | 5, 6 or 7 | 7 |
| 272843 | *Nt5c1b* | 70881 | 9 | 12 |
| 272760 | *1700031M16Rik* | 73302 | 8 | 15 |
| 272723 | *Ms4a13* | 73466 | 8 | 19 |
| 272520 | *1700034O15Rik* | 76606 | 2 | 6 |
| 272519 | *1700013G24Rik* | 69380 | 2* | 4 |
| 272119 | *4931432M23Rik* | 70993 | 5 | 8 |
| 271285 | *4930567K20Rik* | 78056 | 3 | 10 |
| 268996 | *4930583C14Rik* | 75886 | 5 | 10 |
| 264651 | *Tcp10a* | 21460 | 1 | 17 |
| 263708 | *1700016M24Rik* | 69439 | 23 | 15 |
| 263706 | *Slco6b1* | 67854 | 15 | 1 |
| 263615 | *Spesp1* | 66712 | 2 | 9 |
| 258935 | *4930403N07Rik* | 73936 | 11 | 12 |
| 252811 | *4930408G06Rik* | 68179 | 7 | 1 |
| 252761 | *Tbc1d21* | 74286 | 4 or 11 | 9 |
| 252738 | *1700054F22Rik* | 73398 | 7 | 4 |
| 252733 | *1700023A16Rik* | 69371 | 10 | 6 |
| 251434 | *Actl7b* | 11471 | 1 | 4 |
| 250447 | *1700027A15Rik* | 69449 | 2 | 1 |
| 246123 | *LOC622019* | 622019 | 1 | 4 |
| 235535 | *1700001J11Rik* | 72224 | 1 | 9 |
| 233827 | *1700063I17Rik* | 68223 | 3 | 7 |
| 233629 | *4931417E11Rik* | 66740 | 1 | 6 |
| 232593 | *Cmtm2b* | 75502 | 4 | 8 |
| 212793 | *1700049L16Rik* | 108950 | 1 | 10 |
| 212749 | *4930526D03Rik* | 277496 | 4 | 2 |
| 206798 | *Tnp2* | 21959 | 2 | 16 |
| 204664 | *Gm24* | 195236 | 2 | 13 |
| 196313 | *Spata3* | 70060 | 2 or 4 | 1 |
| 195831 | *Cetn1* | 26369 | 2* | 18 |
| 195732 | *4931406B18Rik* | 74054 | 8 | 7 |
| 189668 | *Ubqlnl* | 244179 | 1 | 7 |
| 189648 | *Lypd4* | 232973 | 5 | 7 |
| 189390 | *Cstl1* | 228756 | 4 | 2 |
| 179144 | *Ftmt* | 67634 | 1 | 18 |
| 177847 | *1700016P04Rik* | 69413 | (2<) | 6 |
| 173395 | *Dpep3* | 71854 | 10 | 8 |
| 159541 | *1700020C07Rik* | 75642 | 2 | 2 |
| 159404 | *4933406K04Rik* | 71033 | (8<) | 12 |
| 159193 | *Tktl2* | 74419 | 1 | 8 |
| 159141 | *1700016H13Rik* | 74218 | 4 | 5 |
| 159067 | *4930404H21Rik* | 73808 | 4 | 7 |
| 158494 | *4921511H03Rik* | 70920 | 3* | 5 |
| 158267 | *1700012P22Rik* | 69364 | 4 | 4 |
| 158148 | *Lyzl1* | 67328 | 5 | 18 |
| 158125 | *4921520G13Rik* | 66729 | 3 | 5 |
| 157047 | *4933417A18Rik* | 66761 | 6 | 13 |
| 152656 | *Hsfy2* | 71066 | 1 | 1 |
| 148848 | *4921511C04Rik* | 70853 | 10 | 1 |
| 144202 | *1700008P20Rik* | 69301 | 1 | 7 |
| 143802 | *Tssk3* | 58864 | 2 | 4 |
| 143764 | *Pdcl2* | 79455 | 6 | 5 |
| 141471 | *Galntl5* | 67909 | 9 | 5 |
| 141451 | *Iqcf3* | 68265 | 8 | 9 |
| 141235 | *1700003P14Rik* | 75890 | (4<) | 13 |
| 140115 |  |  | (1) | 17 |
| 138490 | *1700067P10Rik* | 68224 | 3 | 17 |
| 116957 | *Zswim2* | 71861 | 9 | 2 |
| 116871 | *1700094C09Rik* | 78634 | 7 | 8 |
| 116803 | *Hrasls5* | 66727 | 6 | 19 |
| 116705 | *1700057K13Rik* | 73435 | 9 | 1 |
| 116682 | *Iqcf1* | 74267 | 2 or 3 | 9 |
| 108632 | *1700006E09Rik* | 75437 | 6 | 11 |
| 107718 | *H1fnt* | 70069 | 1 | 15 |
| 89197 | *Theg* | 21830 | 7 or 8 | 10 |
| 87748 | *Akap3* | 11642 | 6 | 6 |
| 87624 | *BC048651* | 330277 | 7 | 6 |
| 87446 | *1700022A21Rik* | 72252 | 2* | 5 |
| 87419 | *1700112C13Rik* | 74306 | 5 | 9 |
| 87382 | *4933413B09Rik/Sept12* | 71089 | 8 | 16 |
| 87357 | *Txndc8* | 67402 | 6 | 4 |
| 87326 | *4930479M11Rik* | 74927 | 2 | 7 |
| 87321 | *Catsper1* | 225865 | 12 | 19 |
| 87065 |  |  | (3<) | 1 |
| 87057 | *1700065I17Rik* | 67343 | 3 | 18 |
| 85045 | *4930547C10Rik* | 68274 | 2* | 4 |
| 84927 | *Allc* | 94041 | 12 | 12 |
| 84567 | *4933414I15Rik* | 74408 | (1) | 11 |
| 84522 | *1700023L04Rik* | 76419 | 3 | 6 |
| 84502 | *Stk22s1* | 22116 | 11 | 7 |
| 84435 | *Rshl1* | 83434 | 6 | 7 |
| 84041 | *4930455B14Rik* | 74859 | (4<) | 14 |
| 83967 | *1700025I17Rik* | 70372 | (7<) | 13 |
| 82820 | *Defb19* | 246700 | 2 | 2 |
| 81009 | *EG380907* | 380907 | 2 | 14 |
| 79179 | *1700011K15Rik* | 75456 | 1 | 12 |
| 78654 | *4930524B15Rik* | 67592 | 5 | 11 |
| 78341 | *4933402N03Rik* | 233918 | 3 | 7 |
| 76826 | *Odf4* | 252868 | 5 | 11 |
| 75066 | *4930558C23Rik* | 67654 | (2<) | 3 |
| 75060 | *4921530L21Rik* | 66732 | 2* | 14 |
| 74952 | *4933427I04Rik* | 664620 | (2<) | 4 |
| 73222 | *1700061J05Rik* | 73376 | 6 | 15 |
| 72970 | *4930563D23Rik* | 75328 | 1 | 16 |
| 72491 | *1700025F22Rik* | 69416 | 7 | 19 |
| 67677 | *Kif2b* | 73470 | 1 | 11 |
| 67628 | *Kctd19* | 279499 | 15 | 8 |
| 67403 | *Adam24* | 13526 | 2* | 8 |
| 67392 | *1700123O12Rik* | 73624 | 3 | 4 |
| 67234 | *1700090G07Rik* | 78469 | 4 | 17 |
| 65550 | *1700129C05Rik* | 67932 | 5 | 14 |
| 65488 | *1700008A04Rik* | 69351 | 4 | 11 |
| 63867 | *1700028O08Rik* | 69461 | (3<) | 7 |
| 63799 | *Iqcf5* | 75470 | 2 | 9 |
| 61148 | *4933417M04Rik* | 619288 | 1 | 1 |
| 60992 | *Prdx6-rs1* | 320769 | 1 | 2 |
| 60809 | *4930504O13Rik* | 403200 | 4 | 11 |
| 60688 | *Ccdc70* | 67929 | 2* | 8 |
| 56514 | *4921509E07Rik* | 70897 | 7 | 12 |
| 56430 | *4921510H08Rik* | 66716 | 1 | 10 |
| 56404 | *Odf3* | 69287 | 7 | 7 |
| 55982 | *Ubl4B* | 67591 | 1 | 3 |
| 55891 | *1700011H22Rik* | 69314 | 7 | 4 |
| 55870 | *4921521K07Rik* | 70902 | 2* | 5 |
| 55827 |  |  | (3<) | 10 |
| 54306 | *1700020N01Rik* | 67692 | 2 | 10 |
| 52550 | *1700016G05Rik* | 67690 | 6 | 6 |
| 50108 | *1700011E24Rik* | 75467 | 6 | 17 |
| 46175 | *Hspb9* | 75482 | 1 | 11 |
| 46167 | *Cst13* | 69294 | 4 | 2 |
| 46166 | *Cdrt4* | 66338 | 2 | 11 |
| 46159 | *Rnf151* | 67504 | 4 | 17 |
| 46158 | *Cypt4* | 235067 | 1 | 9 |
| 46146 | *1700054O13Rik* | 67334 | 1 | X |
| 46144 | *1700003H04Rik* | 384775 | 6 | 3 |
| 46140 | *1700008K24Rik* | 69311 | (3<) | 17 |
| 46136 | *1700029H14Rik* | 66501 | 9 | 8 |
| 46132 | *1700021F07Rik* | 72221 | 4 | 2 |
| 46121 | *1700019M22Rik* | 69423 | 1 | 12 |
| 46117 | *1700025E21Rik* | 75647 | 3 | 6 |
| 46114 | *4922502D21Rik* | 381816 | 5 | 6 |
| 46095 | *Cypt3* | 69361 | 2 | X |
| 45833 | *Spata4* | 69281 | 6 | 8 |
| 45824 | *Spata19* | 75469 | 7 | 9 |
| 45821 | *1700030E15Rik* | 75667 | 4 | 9 |
| 45820 | *4930522H14Rik* | 67646 | 5 | 4 |
| 45616 | *1700013N18Rik* | 73318 | 1 | 5 |
| 45607 | *Spaca4* | 69363 | 1 | 7 |
| 45466 | *1700024P04Rik* | 69382 | 1 | 13 |
| 45462 | *1700007E06Rik* | 75459 | 3 | 15 |
| 45442 | *1700042G07Rik* | 67323 | (2<) | 4 |
| 45377 | *1700010M22Rik* | 66328 | 1 | 2 |
| 45306 | *Zdhhc25* | 70073 | 1 | 15 |
| 45302 | *Cypt5* | 66742 | 2 | X |
| 42733 | *Prm1* | 19118 | 2 | 16 |
| 41991 | *D0H6S2654E* | 108161 | (2<) | 13 |
| 38543 | *1700018C11Rik* | 75524 | 5 | 4 |
| 34841 | *Plac8l1* | 69401 | 4 | 18 |
| 33864 | *4930578I06Rik* | 67750 | 5 | 14 |
| 33648 | *2410116G06Rik* | 68236 | 2* | 2 |
| 33646 | *1700054H16Rik* | 432536 | 1 | 11 |
| 33631 | *1700015E13Rik* | 76925 | 2 | 1 |
| 33629 | *Spag4l* | 76407 | 12 | 2 |
| 32242 | *Gykl1 (Gk-rs1)* | 14625 | 1 | 18 |
| 32231 | *4922501K12Rik* | 105511 | 1 | 14 |
| 32213 | *1700018C11Rik* | 75524 | 5 | 4 |
| 31708 | *Fscn3* | 56223 | 7 | 6 |
| 31415 | *1700110M21Rik* | 76627 | (1) | 14 |
| 30959 | *Actl7a* | 11470 | 1 | 4 |
| 30958 | *Actrt2(Arpm2)* | 73353 | 1 | 4 |
| 30482 | *Hils1* | 54388 | 1 | 11 |
| 27027 | *1700019N19Rik* | 67507 | 6 | 19 |
| 27023 | *Cypt12* | 75439 | (1) | 3 |
| 26988 | *Spaca3* | 75622 | 5 or 6 | 11 |
| 26987 | *4932702K14Rik* | 67543 | 1 | 17 |
| 26654 | *Fabp9* | 21884 | 4 | 3 |
| 23887 | *1700113O17Rik* | 68231 | 1 | 2 |
| 23534 | *Tekt3* | 71062 | 8 | 11 |
| 23520 | *Spz1* | 79401 | 1 | 13 |
| 23516 | *1700080E11Rik* | 73532 | 3 | 9 |
| 23515 | *Ropn1* | 76378 | 6 | 16 |
| 23510 | *1700012A03Rik* | 76382 | 3 | 6 |
| 23377 | *Tex22* | 75671 | 3 | 12 |
| 23366 | *1700023F02Rik* | 69433 | (3<) | 10 |
| 23349 | *BC051628* | 332713 | 3 | 2 |
| 21005 | *Cst9* | 13013 | 3 | 2 |
| 12817 | *Capza3* | 12344 | 1 | 6 |
| 10725 | *Tesp1* | 21755 | 5 | 1 |
| 7411 | *Hdgfl1(Pwwp1)* | 15192 | 1 | 13 |
| 4223 | *Pdha2* | 18598 | 1 | 3 |
| 2688 | *Meig1* | 104362 | 3 | 2 |
| 717 | *Pgk2* | 18663 | 1 | 3 |
| 660 | *Cox8c* | 75483 | 2 | 12 |

^a^Unigene ID number (*Mus musculus*, Unigene build #159).

^b^Number of exons based on GenBank database. Presumptive number of exon was represented in parentheses. Asterisks indicate that open reading frame is encoded in a single exon
