# Supplementary material for: Distinct DNA Methylation Dynamics of Spermatogenic Cell-Specific Intronless Genes Is Associated with CpG Content
Source: PLoS One. 2012 Aug 27;7(8):e43658. doi: 10.1371/journal.pone.0043658 (PMC3428356; doi:10.1371/journal.pone.0043658)
Supplement: Table S2 — Primer sequence and parameters for RT-PCR. (DOC) [file pone.0043658.s005.doc]

**Table S2.** Primer sequences and parameters for RT-PCR

| **Gene** | **Primer** | | **Sequence (5’ to 3’)** | **Annealing temp.** | **PCR cycles^a^** |
| --- | --- | --- | --- | --- | --- |
| 4933415F23Rik | 15F23/F | | CTGCTGAGTTGGACATCATCAG | 56 | 25 |
|  | 15F23/R | | ACGATATCCTGGCTTGAGCACA |  |  |
| Ccdc54 | Ccdc54/F2 | | TCCGATGTCATGTGTCTCAGCA | 56 | 25 |
|  | Ccdc54/R2 | | CAGGGCTTCCATCTGATTCTGA |  |  |
| Ccin | Ccin/F | | TCTGGCATTCGTGACAACTTCC | 56 | 25 |
|  | Ccin/R | | TGAAATCTGCTCAGTGGTGCCA |  |  |
| 1700009J07Rik | 09J07/F | | AGCCTCTGACTTATGATCTCAC | 56 | 30 |
|  | 09J07/R | | AGATGTCCTACCTCAACGCTAG |  |  |
| Tssk1 | Tssk1/F | | CGGGATGATAGTGGTCGACTGA | 56 | 25 |
|  | Tssk1/R | | CCTGACCACCTGCAAAGTATCT |  |  |
| Csl | Csl/F | | AGGCCATTGACTCTAAGCTGGA | 56 | 25 |
|  | Csl/R | | CTGACCAACCAGCTTGAACATG |  |  |
| 4922505E12Rik | 05E12/F | | AGCTGGACGAGACGGTTATATC | 56 | 35 |
|  | 05E12/R | | TCTTGTGACCCGCTATCTCCAG |  |  |
| Pbp2 | Pbp2/F | | ATGACATCAGCAGTGGGAACGT | 56 | 25 |
|  | Pbp2/R | | TAACGAGTCCATCACAGTGCCA |  |  |
| Prm3 | Prm3/F2 | | TCCTCCATGAAGAAGCTCGTGG | 56 | 40 |
|  | Prm3/R | | AATGTCCTCTGGCGTGGCAATG |  |  |
| Gm443 | Gm443/F | | ACTGACAGAAGCCTTGCTGGAG | 56 | 25 |
|  | Gm443/R | | AGACCACTGCCACGTTAATGTC |  |  |
| 1700047L15Rik | 47L15/F | | TAACATGGAGGACACGACTGAG | 56 | 25 |
|  | 47L15/R | | TGAAGTGACCGGTCTCTACTGA |  |  |
| Actl7b | Actl7b/F | | GGACTGGGATTGTATCCAGAAC | 56 | 30 |
|  | Actl7b/R | | TAGTCCACATGCAGCTCGTCTA |  |  |
| LOC622019 | 622019/F | | TTGAAGACCAACTTCTGCCAGG | 58 | 35 |
|  | 622019/R | | AAATCAGAGTGTACGTGCGTGC |  |  |
| 1700001J11Rik | 01J11/F | | TGAGAATGACATAAACGTAGGC | 56 | 25 |
|  | 01J11/R | | CCTCCTGTTGCTCCCTTGGAAT |  |  |
| 4931417E11Rik | 17E11/F | | TACTTGTGAGCAATCCTGTCGG | 56 | 25 |
|  | 17E11/R | | GAGCAGATCTTGCTCAAGTTGC |  |  |
| 1700049L16Rik | 49L16/F | | AACTCGCCAACGTCTGAATCCA | 56 | 35 |
|  | 49L16/R | | TCCAATGACAGCAAGGCCTATG |  |  |
| Ubqlnl | Ubqlnl/F | | ATAGCAGCATGGTGTGCCAATC | 56 | 25 |
|  | Ubqlnl/R | | CTTGTTCATCCAGAAGAGCTGT |  |  |
| Ftmt | Ftmt/F | | CATGGCTTACTACTTCTCCAGG | 56 | 25 |
|  | Ftmt/R | | CGTTAACTCCATCCAGGTCTTG |  |  |
| Tktl2 | Tktl2/F | | ACAACTTGGACAATCTCGTGGC | 54 | 30 |
|  | Tktl2/R | | ATTGGCCCAATTTAGCCAGAGC |  |  |
| Hsfy2 | Hsfy2/F2 | | TCTGTGCCTCAGAGCCAGATGA | 56 | 40 |
|  | Hsfy2/R2 | | CTTGACCAAGTCTGGTCTGAAG |  |  |
| 1700008P20Rik | 08P20/F | | CAGGAGGTGTTGAACAAGTGCG | 58 | 30 |
|  | 08P20/R | | AAGTCCTCGAAGGTGATGCCCT |  |  |
| H1fnt | H1fnt/F | | GCGCAGAACTTACGATCCAGAT | 58 | 30 |
|  | H1fnt/R | | TTGAAGAGGCTCTTGACCTGGT |  |  |
| 1700011K15Rik | 11K15/F | | AATGCCTGCAAGATCGCTTCAG | 52 | 30 |
|  | 11K15/R | | GATGCCATGAGTCAAGATGGCA |  |  |
| 4930563D23Rik | 63D23/F | | AAGTCCTACGGCCATGAACTCA | 58 | 25 |
|  | 63D23/R | | GTACCCTCCACTGTGCTAGAGT |  |  |
| Kif2b | Kif2b/F | | ACCAAGTGGATTGCGATGATCC | 58 | 25 |
|  | Kif2b/R | | CATTGGATGCCTTGTCGTCGAA |  |  |
| 4933417M04Rik | 17M04/F | | GTTCAACGGCTTGTTCGACACC | 58 | 25 |
|  | 17M04/R | | AGGTACTGGTGTTACTGTCCAC |  |  |
| Prdx6-rs1 | Prdx6/F | | AGGATGCTAACAGCATGCCTCT | 56 | 25 |
|  | Prdx6/R | | TGACCCAGGAGTATAGCAAGTG |  |  |
| 4921510H08Rik | 10H08/F | | CTTGGTTTCAACCTTCCAGAGG | 56 | 30 |
|  | 10H08/R | | GCATCATTGGTAGTGGAGTCTC |  |  |
| Ubl4b | Ubl4b/F | | CTACTAGGAGCTGTGACAGAGT | 58 | 25 |
|  | Ubl4b/R | | AAGCTGCTGTTGTGCTAGGAGC |  |  |
| Hspb9 | Hspb9/F | | AGCAGTTGCACTCTCGGATGCA | 56 | 25 |
|  | Hspb9/R | | CGCTAAGAGACTCTAAGGATTC |  |  |
| Cypt4 | Cypt4/F | | GATCGAAGCTTAAGAAGAAGCG | 56 | 30 |
|  | Cypt4/R | | CTGATGTTTCATGGGTCTTTCT |  |  |
| 1700054O13Rik | 54O13/F | | AACAGTGTCTTCAGAGAGCCAG | 56 | 30 |
|  | 54O13/R | | AGCCTTCATGGTTCTAGCTGAG |  |  |
| 1700019M22Rik | 19M22/F | | CCATTGTGCAAGGTGGGTTCAT | 58 | 30 |
|  | 19M22/R | | GTTTAGCATTGCTCAGCTGCCA |  |  |
| 1700013N18Rik | 13N18/F | | GCCAAATGATAGTCCCTATCAG | 58 | 25 |
|  | 13N18/R | | CAACATGCAGCGGTACTGTATC |  |  |
| Spaca4 | Spaca4/F | | AGGAGGTGTTCCAGGAGGTCAC | 58 | 30 |
|  | Spaca4/R | | AGTTCTTGTGGGTCACAGCCAG |  |  |
| 1700024P04Rik | 24P04/F | | GCCCACCTTCAAAAGACAATGC | 56 | 25 |
|  | 24P04/R | | AATGAATGAATCTGTGGACGGC |  |  |
| 1700010M22Rik | 10M22/F | | ATAGGCCTCAGACTTGCCAGTC | 56 | 25 |
|  | 10M22/R | | TTAGCCCACTCTCTGAAGATCC |  |  |
| Zdhhc25 | Zdhhc25/F | | ACCATCATGATGCTAGCTCTTG | 56 | 30 |
|  | Zdhhc25/R | | GTTGTCTTCACCAATGCAGTTG |  |  |
| 1700054H16Rik | 54H16/F | | TCTAGAACAGGGAGCACGCATT | 58 | 30 |
|  | 54H16/R | | AACTGGGCTAGAATGTGTGGCC |  |  |
| Gykl1 | Gykl1/F | | AGCGGGAAACTACGATAGTCTG | 58 | 35 |
|  | Gykl1/R | | GACATCCAGATATTGGCACACC |  |  |
| 4922501K12Rik | 01K12/F | | TGAGGAGGAATGAGTCATCTCC | 56 | 40 |
|  | 01K12/R | | CAGCTGAAGCCATCTTGGATGC |  |  |
| Actl7a | Actl7a/F | | TGCGCCATGGCATCATTGTGGA | 58 | 25 |
|  | Actl7a/R | | CAGGCAGGGTATAATGGATCTC |  |  |
| Actrt2 | Actrt2/F | | GAATGGGAACTGGGTGTGAAGC | 58 | 25 |
|  | Actrt2/R | | CAACTGGTCTGGTGAGAACAGG |  |  |
| Hils1 | Hils1/F | ATCCTGGAGTATCTAGCACCTG | | 56 | 25 |
|  | Hils1/R | TAGCAATTGGCATGGTTCAGAC | |  |  |
| 4932702K14Rik | 02K14/F | GTAGAGACAGGCAGGCTGAACT | | 56 | 25 |
|  | 02K14/R | TACGTAGAGTGGTTTGGTGGCC | |  |  |
| 1700113O17Rik | 13O17/F | GTTATTTGGAGAGCCTGGTACC | | 58 | 25 |
|  | 13O17/R | AGCTAGTAGGACATTGGAGCTG | |  |  |
| Spz1 | Spz1/F | TGTCAGAACTTAACGTCAGAGG | | 58 | 25 |
|  | Spz1/R | CTGACATTCATTCTCTAGCAGG | |  |  |
| Capza3 | Capza3/F | TCACCACAATGTGATGGGTGAC | | 56 | 35 |
|  | Capza3/R | GGCTTCGTTAGACAACTCCTGT | |  |  |
| Hdgfl1 | Hdgfl1/F | TGCCAAGTTAAAGGGCTATGCC | | 58 | 30 |
|  | Hdgfl1/R | AGGGTGCTTCTCTTGCACATGC | |  |  |
| Pdha2 | Pdha2/F | ATTGTTGCATCAGAGGAGCTCG | | 56 | 25 |
|  | Pdha2/R | CGAGAATGGACTTCACGGACAG | |  |  |
| Pgk2 | Pgk2/F | GCCAACCCAGATAATGGGTCTA | | 56 | 25 |
|  | Pgk2/R | GGAAGCACCAATCTGCATGTTC | |  |  |
| β-actin | MBA1 | GTGGGCCGCCCTAGGCACCA | | 58 | 25 |
|  | MBA2 | TGGCCTTAGGGTTCAGGGGG | |  |  |

^a^PCR amplification was also investigated at the 3 and 6 cycles before the final round cycles in Figure 2.
