# Supplementary material for: Distinct DNA Methylation Dynamics of Spermatogenic Cell-Specific Intronless Genes Is Associated with CpG Content
Source: PLoS One. 2012 Aug 27;7(8):e43658. doi: 10.1371/journal.pone.0043658 (PMC3428356; doi:10.1371/journal.pone.0043658)
Supplement: Table S3 — Primer sequence and parameters for bisulfite PCR. (DOC) [file pone.0043658.s006.doc]

**Table S3.** Primer sequences and parameters for bisulfite PCR.

| **Gene** | **Primer** | **Sequence (5’ to 3’)** | **Annealing temp.** | **PCR cycles** |
| --- | --- | --- | --- | --- |
| Hdgfl1 | Bis-Hdgfl1/F | AGTTATTGGTTTTTAGAGATAATGTT | 52 | 30 |
|  | Bis-Hdgfl1/R | AACTTCTCTTTAAACTCCTCATAAA |  |  |
| Actl7b | MCT12-5 | TTAGAGATGGGTATTATATG | 55 | 35 |
|  | MCT12-3 | TAAACCATAAACTACTCAAC |  |  |
| Pdha2 | Bis-Pdha2/F | GAATTGTTATAATGGGTAAAGAAGTT | 56 | 33 |
|  | Bis-Pdha2/R | ACATCTTAACATTTATTAAAAAATATA |  |  |
| Kif2b | Bis-Kif2b/F | TTATGTTTATTGGTAGGGTAAAATT | 56 | 30 |
|  | Bis-Kif2b/R | AAAACAAAACTAACTAACCATAATA |  |  |
| Pgk2 | Pgk245 | GTTAAGTTGATTTTGGATAAAGTGGATT | 60 | 35 |
|  | Pgk243 | TAAACCCATTATCTAAATTAACACAAACTTA |  |  |
| 1700049L16Rik | Bis-49L16/F | ATAAGGTTAGAGGTTATGATATAAGT | 52 | 30 |
|  | Bis-49L16/R | CTCCTCCTAATAACTTCATAAATCTA |  |  |
| 4922505E12Rik | Bis-05E12/F | TAAGTTGGAGTTTTTGGTTTGGAGT | 60 | 30 |
|  | Bis-05E12/R | CCTACTCTCCAACTAACAAAAATTC |  |  |
| Ccin | Bis-Ccin/F | GAGGATTGTGATTTGAAAGGTTTAG | 60 | 30 |
|  | Bis-Ccin/R | ATCCACAATCAAAACCATATCCCAA |  |  |
| Ftmt | Bis-Ftmt/F | TTAGAATTTTGTGTTAGTAATTTAGT | 52 | 35 |
|  | Bis-Ftmt/R | CAATACAAAACTAATATACTTA |  |  |
| Tktl2 | Bis-Tktl2/F | GTTGGGATATTGGGATATTGTAAAAT | 52 | 30 |
|  | Bis-Tktl2/R | TAACCCTAATTAAATAAATTC |  |  |
| Pbp2 | Bis-Pbp2/F | GGTTGTAGAGTTTGTGTGGTTA | 56 | 35 |
|  | Bis-Pbp2/R | TATATCAAAACCATCCCATAAAATA |  |  |
| Prdx6-rs1 | Bis-Prdx6/F | ATGGGATTTGATATTTTTAGAGATT | 56 | 30 |
|  | Bis-Prdx6/R | TTATCATTACATTTACTAATCCCTA |  |  |
| H1fnt | Bis-H1fnt/F | TTATATATTTGTAGGTGTGAAGAGT | 56 | 30 |
|  | Bis-H1fnt/R | TCAAAACTCTCTCTACAAACTACTA |  |  |
| 1700008P20Rik | Bis-08P20/F | TATTGAATTTGGAGAGGAAAGAGTT | 56 | 30 |
|  | Bis-08P20/R | ACAACCTAAACCTCTAATACAACTA |  |  |
| Hspb9 | Bis-Hspb9/F | ATTAGTTTTTAGGATGGAGTTGTAAT | 60 | 30 |
|  | Bis-Hspb9/R | CTTAATTACTCACAACCATTACCCTA |  |  |
| 4921510H08Rik | Bis-10H08/F | TTGTTAGGGTGTGAGTATTGTTTAGT | 56 | 30 |
|  | Bis-10H08/R | ATAAAATATAAAAACCACTCCCAAAT |  |  |
| Capza3 | Bis-Capza3/F | TTTTATAGTTGGTATGGTTTGTTAGT | 56 | 35 |
|  | Bis-Capza3/R | CTAAATAACATAACCAATAACAACAA |  |  |
| 4930563D23Rik | Bis-63D23/F | AGTTTAAATGAAAAGGAATTAGAAAT | 52 | 30 |
|  | Bis-63D23/R | TAAATAACTCACATTTTCCCATCTAA |  |  |
| Actl7a | Bis-Actl7a/F | GGTGTATATTAATTAGAATGTAGAT | 56 | 30 |
|  | Bis-Actl7a/R | TAAAAACCTAATCACTAACTTTCTTA |  |  |
| Ubqlnl | Bis-Ubqlnl/F | TGGGATAGTTGTGAAAGGTTATTAT | 56 | 30 |
|  | Bis-Ubqlnl/R | ACATAAACCTTATAAATAAACAAATA |  |  |
| Oaz1 | 01-52 | TTTTTGGGTTGTTATTGTGG | 59 | 35 |
|  | 01-32 | AAAACAAAACTCTCACCTCT |  |  |
| IAP | Bis-IAP/F | TGATAGTTGTGTTTTAAGTGGTAAAT | 60 | 25 |
|  | Bis-IAP/R | CAAAACTTTATTACTTACATCTTCAA |  |  |
